# Catalytic ozone oxidation of benzene at low temperature over MnOx/Al-SBA-16 catalyst

**DOI:** 10.1186/1556-276X-7-14

**Published:** 2012-01-05

**Authors:** Jong Hwa Park, Ji Man Kim, Mingshi Jin, Jong-Ki Jeon, Seung-Soo Kim, Sung Hoon Park, Sang Chai Kim, Young-Kwon Park

**Affiliations:** 1 Graduate School of Energy and Environmental System Engineering, University of Seoul, Seoul 130-743, South Korea; 2 Department of Chemistry, BK21 School of Chemical Materials Science and Department of Energy Science, Sungkyunkwan University, Suwon 440-746, South Korea; 3 Department of Chemical Engineering, Kongju National University, Cheonan 330-717, South Korea; 4 Department of Chemical Engineering, Kangwon National University, Samcheok 245-711, South Korea; 5 Department of Environmental Engineering, Sunchon National University, Suncheon 540-742, South Korea; 6 Department of Environmental Education, Mokpo National University, Muan 534-729, South Korea; 7 School of Environmental Engineering, University of Seoul, Seoul 130-743, South Korea

**Keywords:** Al-SBA-16, Mn precursors, benzene, ozone, catalytic oxidation

## Abstract

The low-temperature catalytic ozone oxidation of benzene was investigated. In this study, Al-SBA-16 (Si/Al = 20) that has a three-dimensional cubic Im3m structure and a high specific surface area was used for catalytic ozone oxidation for the first time. Two different Mn precursors, i.e., Mn acetate and Mn nitrate, were used to synthesize Mn-impregnated Al-SBA-16 catalysts. The characteristics of these two catalysts were investigated by instrumental analyses using the Brunauer-Emmett-Teller method, X-ray diffraction, X-ray photoelectron spectroscopy, and temperature-programmed reduction. A higher catalytic activity was exhibited when Mn acetate was used as the Mn precursor, which is attributed to high Mn dispersion and a high degree of reduction of Mn oxides formed by Mn acetate than those formed by Mn nitrate.

## Introduction

Hazardous air pollutants [HAPs] are airborne species that are known to or are anticipated to cause adverse effects on human health and environment. HAPs are characterized by their toxicity, carcinogenicity, bioaccumulation, persistence, and dispersion. Most HAPs, however, are not regulated/managed, producing secondary pollutants and odor [1]. Benzene, a representative HAP, is a well-known carcinogen. Long-term exposure to benzene can cause blood dyscrasias such as a decrease in erythrocytes, aplastic anemia, and leukemia [2]. Therefore, in recent years, considerable attention has been paid to the removal of benzene and other HAPs.

Ozone has been widely used for pollution treatment in the semiconductor industry, water treatment, and air cleaning [3-5]. In particular, catalytic ozone oxidation has high pollutant-removal efficiency and low energy consumption [6]. In the catalytic ozone oxidation process, ozone is decomposed into activated oxygen species that can oxidize organic compounds. Recently, researches on the catalytic ozone oxidation of volatile organic compounds [VOCs] including HAPs have been performed [7-9]. The HAP removal process involving ozone addition is economically advantageous because it can be performed at a temperature much lower than that required for conventional HAP removal processes. Thus far, Al_2_O_3_, SiO_2_, and zeolite catalysts impregnated with metal have usually been used for catalytic ozone oxidation. In particular, supports with a large specific surface area have good dispersion of metal oxides within the supports, leading to high reaction activity [4,9].

Recently, mesoporous materials such as MCM-41 and SBA-15 have been widely used as supports for various reactions because of their uniform pores and large specific surface areas. In particular, SBA-16 is expected to exhibit high activity during the catalytic ozone oxidation of benzene because of its super-large cage, large surface area, and high thermal stability. The three-dimensional channel connectivity of SBA-16 makes it even more favorable for mass-transfer kinetics than the other hexagonal mesoporous materials having unidirectional pore structures. To the best of our knowledge, SBA-16 has never been used for the catalytic ozone oxidation of benzene. MnO_x _is a metal oxide that exhibits high activity during the decomposition of VOCs at a low temperature [10]. Therefore, in this study, Al-SBA-16 was impregnated with Mn by using two different Mn precursors, i.e., Mn(CH_3_COO)_2 _(Mn acetate) and Mn(NO_3_)_2 _(Mn nitrate), to investigate the effect of Mn precursors on the catalytic ozone oxidation of benzene.

## Experimental details

### Synthesis of MnO_x_/Al-SBA-16 catalysts

The detailed procedure for the synthesis of mesoporous silica SBA-16 with cubic Im3m structure is described in the literature [11]. A poly(alkylene oxide)-type triblock copolymer, i.e., F127 (EO_106_PO_70_EO_106_, MW = 12,600, Sigma, St. Louis, MO, USA), was dissolved in an aqueous HCl solution, and tetraethyl orthosilicate [TEOS] (98%) was added at 35°C. The solution was stirred for 15 min by a magnetic stirrer at the same temperature. The molar composition of the mixture was F127:TEOS:HCl:H_2_O = 0.0040:1.0:4.0:130. This mixture was put in an oven for 24 h at the same temperature. The mixture was then put in an oven at an elevated temperature of 100°C for 24 h. After this hydrothermal aging, the solid product formed was recovered by filtration and was dried at 100°C without washing. The dried sample was washed with ethanol, dried in an oven at 100°C, and calcined at 550°C. Al incorporation in the sample was performed with an ethanolic solution of AlCl_3 _(Si/Al = 20). After completely evaporating the solvent (ethanol) in a rotary evaporator, the sample was calcined in air at 550°C. The Al-incorporated sample is hereafter referred to as Al-SBA-16.

The amount of Mn impregnated using Mn(NO_3_)_2 _(98%, Aldrich, St. Louis, MO, USA) and Mn(CH_3_COO)_2 _(> 99%, Aldrich, St. Louis, MO, USA) as the Mn precursors was 15 wt.%. The Mn-impregnated material was calcined at 550°C. Al-SBA-16 catalysts synthesized using Mn nitrate and Mn acetate as the Mn precursors are hereafter referred to as Al-SBA-16-MN15% and Al-SBA-16-MA15%, respectively.

### Characterization of MnO_x_/Al-SBA-16

X-ray diffraction [XRD] patterns of the catalyst were obtained using an X-ray diffractometer (D/MAX-III, Rigaku, Akishima, Japan) with Cu-Kα radiation. The N_2 _adsorption-desorption isotherms and the Brunauer-Emmett-Teller [BET] surface area of the catalyst were obtained using an ASAP-2010 apparatus (Micromeritics, Norcross, GA, USA). Temperature-programmed reduction [TPR] analysis was performed using a ChemBET 3000 (Quantachrome, Boynton Beach, FL, USA) setup. X-ray photoelectron spectroscopy [XPS] was performed using an AXIS Nova spectrometer (Kratos Inc., NY, USA). A monochromatic Al Kα (1,486.6 eV) of X-ray source and 40 eV of analyzer pass energy were used under ultra-high vacuum conditions (5.2 × 10^-9 ^Torr).

### Benzene oxidation with ozone

Catalytic reaction experiments were performed in a fixed-bed flow reactor. Ozone was produced from O_2 _using a silent-discharge ozone generator. Before each experiment, the sample was heated at 450°C in a Pyrex glass reactor under an O_2 _flow. The catalyst was then cooled and maintained at 80°C. In each experiment, 0.05 g of the catalyst was used. The ozone flow rate and benzene inlet concentration were set at 120 mL/min and 100 ppm, respectively. The product gas sample was passed through a GC/FID (6000 Series, Young Lin, Anyang, South Korea) with an HP-5 column (Agilent Technologies Inc., Santa Clara, CA, USA) to analyze the benzene conversion, an indoor gas analyzer (ISR-401, WOORI Industrial System Co., Ltd., Chungcheongbuk-do, South Korea) used for the CO and CO_2 _products, and an ozone analyzer (LAB-S, Ozonetech, Daejeon, South Korea) for the ozone conversion. In this study, the gas-phase reaction of benzene with ozone was shown to be negligible.

## Results and discussion

### Characterization of Al-SBA-16

Table 1 lists the textural properties of Al-SBA-16 catalysts impregnated with Mn nitrate and Mn acetate. Al-SBA-16-MN15% had a greater BET surface area than Al-SBA-16-MA15%.

**Table 1:** Textural properties of the catalysts

	*S*_BET_ (m^2^/g)	Pore size (nm)
Al-SBA-16 MA15%	359	4.94
Al-SBA-16 MN15%	489	4.60

**Figure 1:**
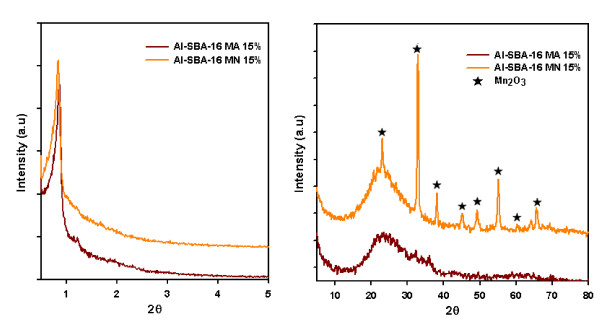
**The powder XRD patterns of Al-SBA-16 with various Mn precursors**.

The XRD pattern of the synthesized SBA-16 is shown in Figure 1, which could be identified as that of a cubic SBA-16 with sharp (110) and small (200) reflections. This result indicates that the cubic mesostructure was not destructed by the incorporation of alumina on the silica framework. It is shown in Figure 1 that the Mn/Al-SBA-16 prepared by using Mn nitrate (Al-SBA-16-MN15%) exhibited high-angle peaks representing Mn_2_O_3 _particles, while the Mn/Al-SBA-16 prepared by using Mn acetate (Al-SBA-16-MA15%) exhibited no Mn-particle peak. This result indicates that Mn was dispersed uniformly within Al-SBA-16-MA15%, whereas it was poorly dispersed in Al-SBA-16-MN15%, and Mn oxides existed as large-sized particles.

**Figure 2:**
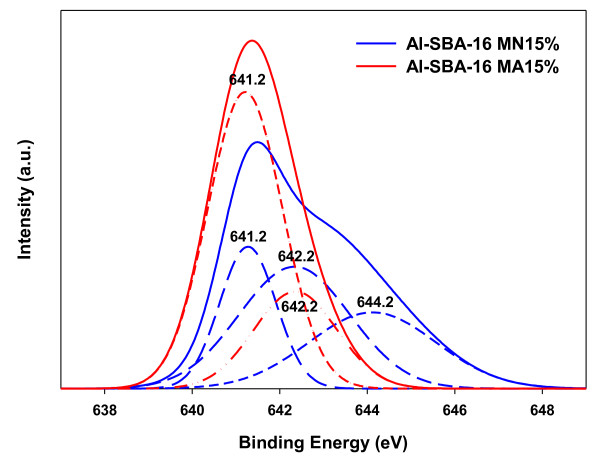
**XPS spectra of Al-SBA-16 with various Mn precursors**.

Figure 2 shows a comparison between the Mn 2p XPS spectra of Al-SBA-16-MA15% and Al-SBA-16-MN15%. The peak for Al-SBA-16-MN15% was divided into three peaks located at 641.2, 642.3, and 644.1 eV obtained by peak deconvolution, representing Mn_2_O_3 _(641.2 ± 0.2 eV), MnO_2 _(642.2 ± 0.4 eV), and Mn nitrate (644.2 ± 0.4 eV), respectively [12]. On the other hand, the peak for Al-SBA-16-MA15% was divided into two peaks located at 641.2 and 642.3 eV, implying the dominance of Mn_2_O_3 _within Al-SBA-16-MA15%. The XRD and XPS results suggested that well-dispersed Mn oxides were formed by Mn acetate, while several different types of poorly dispersed Mn oxides were formed by Mn nitrate. On the basis of these results, it was expected that Al-SBA-16-MA15% would show a higher activity for the catalytic ozone oxidation of benzene than Al-SBA-16-MN15%.

**Figure 3:**
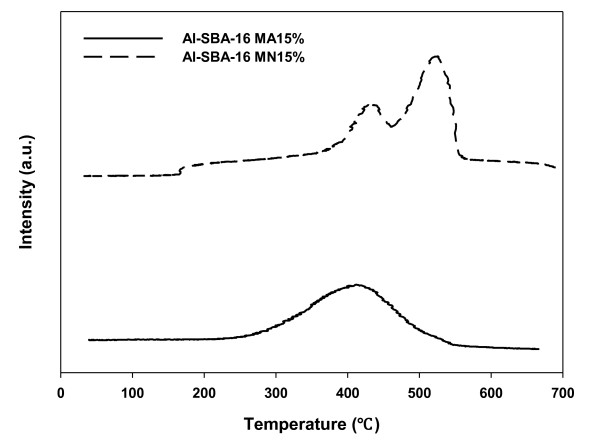
**TPR of Al-SBA-16 with various Mn precursors**.

As shown by the TPR results (Figure 3), Al-SBA-16-MA15% has higher reduction ability than Al-SBA-16-MN15%. This implies that Al-SBA-16-MA15% has higher lattice oxygen mobility, leading to higher activity for the oxidation reaction. In addition, as mentioned above, the order of catalytic activity for the VOC oxidation of Mn oxides is Mn_3_O_4 _> Mn_2_O_3 _> MnO_2 _[13]. In this study, it was shown that highly active Mn_2_O_3 _was dispersed well in Al-SBA-16-MA15%, while Al-SBA-16-MN15% contained large-sized Mn_2_O_3 _particles, resulting in low activity. Moreover, Al-SBA-16-MN15% contained MnO_2 _and Mn nitrate with low activity, which is supposed to be another reason for the low activity of Al-SBA-16-MN15%.

### Benzene oxidation with ozone

**Figure 4:**
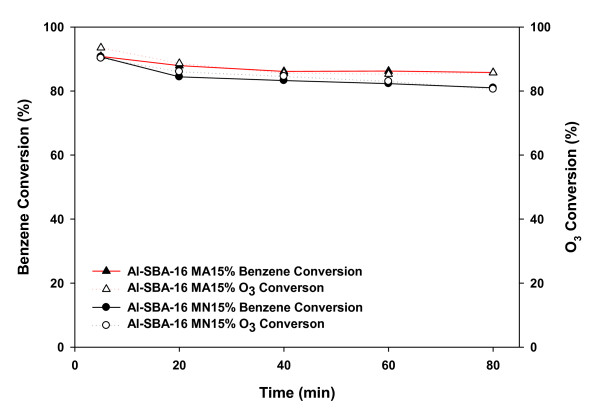
**Benzene and ozone conversions over Al-SBA-16 with various Mn precursors at 80°C**.

Figure 4 shows a comparison between the conversions of benzene and ozone obtained using two Mn-impregnated Al-SBA-16 catalysts. For 80 min, Al-SBA-16-MA15% showed benzene and ozone conversions about 5% higher than those shown by Al-SBA-16-MN15% despite having a low surface area. The conversions reduced with an increasing reaction time for both Al-SBA-16-MA15% and Al-SBA-16-MN15%; however, the extent of reduction was larger for Al-SBA-16-MN15%.

**Figure 5:**
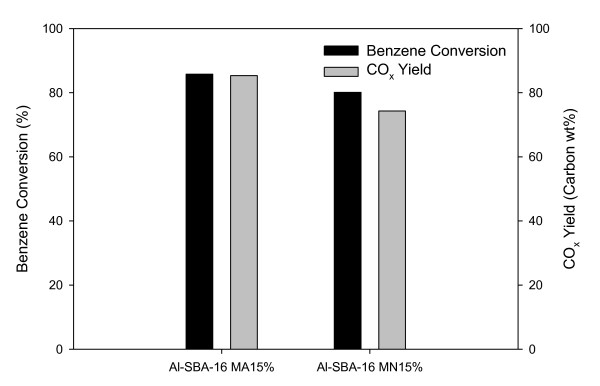
**Effect of Mn precursors on benzene conversion and CO_x _yield over Al-SBA-16**. Time on stream, 80 min; temperature, 80°C.

Figure 5 shows the benzene conversions and yields of CO_x _(CO_2 _+ CO) obtained for 80 min of reaction time using two catalysts. Both the benzene conversion and CO_x _yield were about 85% for Al-SBA-16-MA15%, whereas for Al-SBA-16-MN15%, the CO_x _yield (74%) was significantly lower than the benzene conversion (81%). As shown by the TPR results, Al-SBA-16-MA15% has a higher degree of reduction than Al-SBA-16-MN15%, which may lead to higher lattice oxygen mobility and higher oxidation activity. It has been reported that the order of catalytic activity of Mn oxides for the oxidation of VOCs is Mn_3_O_4 _> Mn_2_O_3 _> MnO_2 _[13]. In this study too, Al-SBA-16-MA15% containing well-dispersed, more-active Mn_2_O_3 _showed a higher activity for benzene oxidation, whereas Al-SBA-16-MN15% containing large-sized Mn-oxide particles, including less-active MnO_2 _and Mn nitrate together with Mn_2_O_3_, showed a lower activity.

**Figure 6:**
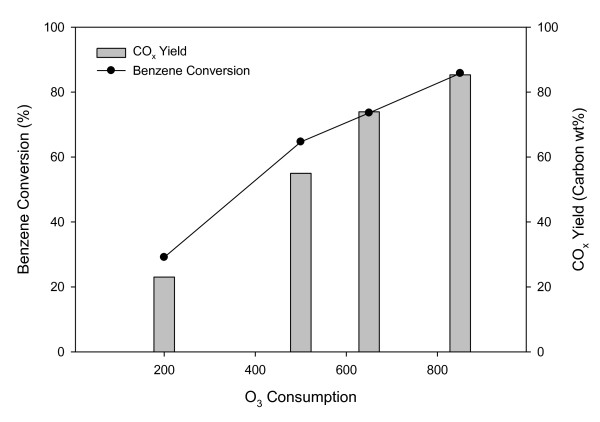
**Effect of ozone consumption on benzene conversion and CO_x _yield over Al-SBA-16 with Mn acetate**. Time on stream, 80 min; temperature, 80°C.

Figure 6 shows the CO_x _yield and benzene conversion obtained by using Al-SBA-16-MA15% for 80 min with different ozone consumptions. It is shown that both the benzene conversion and CO_x _yield increased with ozone consumption. When ozone was not added, virtually, no reaction occurred (data not shown). Ozone is decomposed into oxygen species as a result of interactions with Mn oxide, forming catalytic active sites by the following mechanisms [14]:

(1) $${\text{O}}_{3}\to {\text{O}}_{2}+{\text{O}}^{*}$$

(2) $${\text{O}}^{*}\text{ + }{\text{O}}_{3}\to {\text{O}}_{2}+{\text{O}}_{2}^{*}$$

(3) $${\text{O}}_{2}^{*}\to {\text{O}}_{2}+*$$

where * represents the catalytic active site. The oxygen species formed during the decomposition of ozone oxidize benzene, producing oxygen-containing by-products. These by-products are further oxidized to CO_x_. The fact that a gas-phase reaction between ozone and benzene did not occur indicates that ozone itself does not function as the oxidizer. Rather, ozone is decomposed into oxygen species by the above-shown mechanisms, and these oxygen species oxidize benzene. As shown in Figure 6, the consumption of ozone had a good correlation with the conversion of benzene: a higher benzene conversion was obtained at a higher consumption of ozone.

## Conclusions

Two different Mn precursors were used to synthesize mesoporous catalysts for the catalytic ozone oxidation of benzene by impregnating Al-SBA-16 with Mn. The catalytic activity of Al-SBA-16-MA15% was higher than that of Al-SBA-16-MN15%. It was shown that the type of precursors used for Mn impregnation influenced the dispersion, oxidation state, and oxygen mobility of the impregnated Mn. XRD and TPR analyses showed that Al-SBA-16-MA15% had better Mn dispersion and a higher degree of reduction than Al-SBA-16-MN15%. XPS analysis showed that highly dispersed Mn oxides could form main active sites for Al-SBA-16-MA15%. These catalytic properties appear to have induced the high catalytic activity of Al-SBA-16-MA15%.

## Abbreviations

XPS: X-ray photoelectron spectroscopy; XRD: X-ray diffraction.

## Competing interests

The authors declare that they have no competing interests.

## Authors' contributions

JHP, JMK, MJ, JKJ, SSK, SHP, and SCK participated in some of the studies and in drafting the manuscript. YKP conceived the study and participated in all experiments of this study. Also, YKP prepared and approved the final manuscript. All authors read and approved the final manuscript.
